# The Leader Vitality Scale: Development, Psychometric Assessment, and Validation

**DOI:** 10.3389/fpsyg.2022.884672

**Published:** 2022-06-10

**Authors:** Jamie Shapiro, Stewart I. Donaldson

**Affiliations:** Division of Behavioral & Organizational Sciences, Claremont Graduate University, Claremont, CA, United States

**Keywords:** positive psychology at work, positive organizational psychology, positive leadership, vitality, leadership, work-related well-being

## Abstract

One of the most important units of analysis for positive organizational psychology research is leaders and future leaders in the workplace. Leaders often have a large responsibility for and influence on the well-being and performance of their followers. They also face the unique challenge of serving their followers and the organization while needing to maintain their own vitality and well-being. Vitality can provide a foundation of energy resources to a leader to serve at their full capacity. This study develops and empirically examines a new three factor scale to measure leader vitality which includes physical, psychological, and emotional components. In study 1, a total of 175 participants (including *n* = 128 leaders) completed the Leader Vitality Scale (LVS) and other positive psychology related measures. Exploratory factor analysis and then confirmatory factor analysis showed that the LVS is hierarchical with three distinct factors, with overall vitality as the higher-order factor. Correlational tests with two established vitality scales for general use showed that the LVS is positively related to existing scales, demonstrating convergent validity. In study 2, data was gathered from 92 top level leaders in the C-Suite (*n* = 25), vice presidents (*n* = 23), directors (*n* = 21), and managers (*n* = 23) of organizations across the United States. Results showed that LVS scores significantly correlated with life satisfaction, positive emotions, positive functioning at work, and psychological capital. Overall, these findings suggest that the LVS is a valid measure for assessing leader vitality, and can used in future studies of well-being and positive functioning at work.

## Introduction

Donaldson and Ko (2010) defined positive organizational psychology “as the scientific study of positive subjective experiences and traits in the workplace and positive organizations, and its application to improve the effectiveness and quality of life in organizations.” In their systematic review of the empirical literature, they identified leadership as the most empirically studied topic to date, and one of the most important potential influencers of worker well-being and positive functioning. More recent meta-analyses and systematic reviews show positive organizational psychology interventions that target improving employee, team, and leader well-being can be highly effective for achieving desired workplace outcomes (see Donaldson et al., 2019, 2021a; Donaldson and Chen, 2021). Additional recent research has shown that the PERMA + 4 building blocks of well-being (e.g., positive emotions, engagement, relationships, meaning, achievement, physical health, positive mindset, environment, and economic security) predict for the first time that well-being and positive functioning at work measures (including job and academic performance measures) above and beyond mono-method and self-report bias (Donaldson et al., 2021b, 2022; Donaldson and Donaldson, 2021a). One important potential mechanism for many of the findings related to leadership is that positive organizational psychology interventions lead to higher levels of leader vitality (see Cameron, 2021). However, a valid measure of leader vitality is sorely needed so these potential relationships can be explored with scientific rigor.

Leaders of organizations have continuous demands placed on them from cultivating teams, to building organization culture, to managing to the bottom line, to caring for employee well-being and positive functioning. Resources are required to meet the continuous demands placed on leaders and energy is one of these valuable resources. Vogel (2017) showed through his research in human energy and work that when individuals feel energized and are thriving, leadership capacity is expanded. Human energy is both generative and dynamic and it generally benefits the leader, work teams, and organizations. Vitality is an inner resource that can foster an abundance of energy available to self that can serve leaders in meeting the pressures of their roles. Vitality is defined as the as positive aliveness and having access to the energy within oneself (Ryan and Frederick, 1997). The lens of Conservation of Resources (COR) theory helps to explain that when a leader has energy available to oneself, this protects the leader from depletion. Conservation of Resources scholars consider energy to be a scarce resource, such that it must be replenished when depleted (Hobfoll, 1989; Hobfoll and Shirom, 2001). Leaders need energy resources to serve their followers’ needs and organizations without depleting their own energy in the process or burning out.

Burnout is defined as “emotional/and or physical exhaustion, lowered work productivity, and over depersonalization” (Perlman and Hartman, 1982, p. 293). According to both the Stanford School of Business and Harvard Business School, burnout costs businesses between $125 billion and $190 billion every year in healthcare costs in the United States alone (Garton, 2017). In light of the high potential for leadership burnout, leaders and organizations need a way to support leaders and a deeper understanding of how vitality can be cultivated and utilized to protect leaders from burnout and increase leadership capacity. Emotional labor is a key mechanism in leadership that drains vitality. Emotional labor is defined by the suppression of internal feelings to create the necessary outward expression to elicit the proper state of mind and coordination of others (Gardner et al., 2009). Emotional labor is a continuous requirement for leaders to lead effectively in organizations and it can drain vitality in leaders and lead to burnout (Gardner et al., 2009). Leaders utilize emotional labor to alter their emotional expression in two different ways, surface acting (when leaders change their outward emotional expressions but do not attempt to feel the emotions that they are displaying) and deep level acting (when leaders attempt to feel the emotions they want to display) (Humphrey et al., 2015). Surface level acting has been shown to have the greatest negative impact to leader resources and well-being (Humphrey et al., 2015).

**FIGURE 1:**
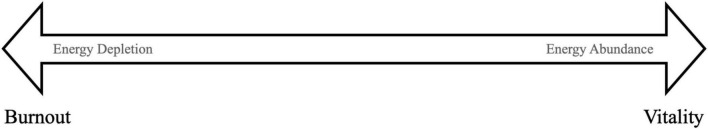
Burnout to vitality.

Burnout and vitality can be seen as opposite ends of a spectrum seen in Figure 1. Vitality is when a leader has an abundance of energy available to self, where burnout is when energy is depleted.

Instead of seeking to understand leader burnout in more depth, this paper focuses on creating a deeper understanding of vitality and how to better define and measure the construct for leaders. “People who are fully vital have honest, trustworthy and generative relationships with those around them (both at home and at work; Cannon, 2011, p.308).” Leaders that have a foundation of vitality have the potential to create a “positive ripple effect” in the organization. The positive leadership ripple effect is based on the theories and empirical research including positive energizers and energy networks theory (Baker et al., 2003; Cameron, 2021) and positive relational energy (Owens et al., 2016). According to Cameron (2021) individuals can be identified as “positive energizers,” or “negative energizers.” The positive ripple effect refers to leaders impacting organizations through being “positive energizers.” Research on energy has shown that the resource of relational energy in an individual is a key mechanism for transferring energy to others through positive relationships (Vogel, 2017; Dutton et al., 2020). A leader’s vitality potentially increases the capacity for more positive relational energy with followers, and therefore can create the positive-energy networks within an organization that help enable a positive climate and enhance performance (Baker et al., 2003; Baker, 2019; Cameron, 2012, 2021). Studies have shown that positively energizing leaders can increase psychological capital and empowerment in followers (Avey et al., 2011) and enhance trust in leadership (Norman et al., 2010). Additionally, positive leaders have been shown to increase employee well-being, life satisfaction and job satisfaction (Kelloway et al., 2013; Zbierowski and Góra, 2014; Cameron et al., 2017). Cameron (2021) showed with a sample of 600 middle and upper-level leaders that energizing behaviors led to higher organizational outcomes including higher productivity, increased quality, better employee morale, higher customer satisfaction and financial strength. Leaders need a foundation of vitality to create the continual transference of positive relational energy to achieve these beneficial organizational outcomes.

### Vitality

Vitality is an inner resource that can foster an abundance of energy available to self. The construct of vitality was first identified in 1997, when Ryan and Frederick (1997) defined the concept of vitality and a developed a scale to measure it called the Subjective Vitality Scale (SVS). The construct of vitality due to is complexness continues to be a concept that is debated with little cohesion on a single definition (Deng et al., 2015). Vitality is often defined and measured through psychological factors alone in psychology through scales like the Subjective Vitality Scale (SVS) (Lavrusheva, 2020). Where the medical field tends to focus more heavily on the emotional and physical factors of vitality with scales like the health-related quality-of-life (HRQOL) and the SF-36 Health Survey that has four questions on vitality (Deng et al., 2015; Lavrusheva, 2020). Vitality traces back to Ancient Greek and Eastern culture and philosophy in concepts like Chi in China, Ki in Japan, Bayu in Indonesia, and Prana in India (Lavrusheva, 2020). The common theme of these ancient concepts of vitality is an “underlying life energy or force flowing through living things” (Lavrusheva, 2020, p. 2).

The current study examines physical, psychological, and emotional vitality. Most definitions of vitality are combinations of these three factors (Richman et al., 2009). For purposes of this study, physical vitality is defined as energy available to oneself or a sense of physical aliveness (Cannon, 2011). Psychological or mental vitality is defined as the mental energy to think clearly, focus, be alert, have flexible thinking, and create a positive outlook (Richman et al., 2009). Emotional vitality is defined as the energy available to oneself to regulate emotions effectively (Penninx et al., 1998). Emotional vitality is closely related to emotional intelligence and a potential an antecedent. A key distinction between the two concepts is emotional intelligence is focused on awareness and management of emotion and emotional vitality can be seen as the energy resource required for that awareness and management (Goleman and Boyatzis (2017). It is believed that these three factors will create a more complete understanding of leader vitality.

### Overview

We conducted two empirical studies to test and validate the LVS. The first study used exploratory factor analyses and confirmatory factor analysis to confirm the LVS questions and factor structure of the scale. In study 2, we directly recruited top level leaders from US based organizations with C-suite to manager level roles to further validate the LVS.

## Study 1

The primary purpose of the first study was to develop a sound measure for leader vitality that encompassed all three factors: physical, psychological, and emotional. We generated questions by reviewing a range of the items from the various vitality scales that have been developed in both the psychology and medical fields. The scale items were first tested for face validity to refine the language of each question. We then tested the scale using exploratory factor analysis (EFA), a method that reduces and refines items, in a sample of leaders collected through convenience sampling methods. The scale was then validated with confirmatory factor analysis. Convergent validity was established through correlating the new scale with the validated Subject Vitality Scale (Ryan and Frederick, 1997) and the SF-36 Health Survey four question vitality scale (RAND Corporation, 2021). We tested the following hypotheses in study 1:

H1: Vitality is a higher order construct that has three sub-factors including physical, psychological, and emotional vitality.

H2: The three sub-factors of leader vitality will be positively correlated with one another.

H3: The three sub-factors of vitality are correlated but measure distinct aspects of leader vitality.

## Method

### Scale Development

The first step of developing the questions for the Leader Vitality Scale (LVS) was to evaluate the existing validated scales in vitality including the Subjective Vitality Scale (Ryan and Frederick, 1997), SF-36 Health Survey (RAND Corporation, 2021), Vitality Measure (Andersen and Lobel, 1995), and the Emotional Vitality Measure (Penninx et al., 1998). These existing scales were studied to determine the original 15 questions for the LVS that we categorized by physical vitality, psychological or mental vitality, and emotional vitality. There were a total of five questions selected or created for each category. The questions were first tested for face validity with a sample of 12 professionals with both academic and professional backgrounds. The questions were modified based on recommendations and finalized. The response set for each question was a 7-point Likert-type scale ranging from strongly disagree (1) to strongly agree (7).

### Participants

A total of 175 people participated in the survey and were recruited through LinkedIn and Facebook. The reason that both Facebook and LinkedIn were utilized was to obtain a predominately workforce-based sample of leaders. The sample of *N* = 175 contained a total of 73% of participants (*n* = 128) that self-identified as leaders in their organization and 85% of participants (*n* = 149) who are working full or part-time. Of the sample, 72% were female (*n* = 126) and 26% were male (*n* = 49). The participants’ ages ranged from 18 to 70 + years, with the highest number of respondents being between 41 and 55 years (*n* = 93). In terms of education, 89% of the participants possessed a bachelor’s degree or higher (*n* = 155). Regarding race/ethnicity, the majority self-identified as White/Caucasian (87%, *n* = 153), followed by 4% Hispanic/Latino (*n* = 7), Multiracial (3%, *n* = 5), Asian (2%, *n* = 4), and African American/Black (2%, *n* = 3).

### Procedure

Participants completed a 65-question survey that included the four vitality questions from the SF-36 Health Survey (RAND Corporation, 2021), the Satisfaction with Life Satisfaction Scale (SWLS) (Diener et al., 1985), the 15 question Leader Vitality Scale (LVS), the six question Subjective Vitality Scale (SVS) (Ryan and Frederick, 1997), the Psychological Capital PCQ-12 (Luthans and Youssef-Morgan, 2017), and three questions utilizing Cantril’s Ladder (Gallop, 2021). The three questions that utilized Cantril’s ladder included asking participants to rate on a scale of 1–10 the three factors of vitality. Demographic information was collected at the end of the survey, including age, gender, marital status, ethnicity, education level, employment status, income, leadership status and management status. All questions in the survey were required to be answered. Participants did not receive any compensation for completing the survey.

### Measures

#### SF-36 Health Survey

The SF-36 health survey is a health survey with only 36 questions used around the world. It is a generic measure of health status that includes four questions in vitality. Participants were asked to answer the four vitality questions based on how they feel and how things have been with them during the last 4 weeks on a scale from 1 (all of the time) to 6 (none of the time), with two questions being reverse coded. The questions included, “Did you feel full of pep?”, “Did you have a lot of energy?”, “Did you feel worn out?”, and “Did you feel tired?”

#### Satisfaction With Life Scale

Participants were asked to rate life satisfaction utilizing the Satisfaction with Life Scale (SWLS) which is a well validated and widely used scale of subjective well-being (Diener et al., 1985). This scale consists of five questions, “In most ways my life is close to my ideal,” “The conditions of my life are excellent,” “I am satisfied with my life,” “So far I have gotten the important things I want in life,” “If I could live my life over, I would change almost nothing.” The scale uses a 7-point Likert scale from “strongly disagree” to “strongly agree.”

#### Leader Vitality Scale

Participants rated themselves on a 15 item three-factor scale with five questions for physical vitality, five questions for psychological or mental vitality and five questions for emotional vitality. The response set was a 7-point Likert scale ranging from strongly disagree (1) to strongly agree (7). The 15 initial questions are shown in Table 1.

**TABLE 1:** Initial 15 Items of the LVS.

	Factor Name and Items
	**Factor 1: Physical vitality**
PHY1	I drink water throughout the day
PHY2	I regularly eat healthy
PHY3	I incorporate movement into my day
PHY4	I have the physical stamina to do the things I want to do in my life
PHY5	I feel well rested when I wake up in the morning
	Factor 2: Psychological vitality
PSY1	I feel alive and vital
PSY2	I nearly always feel awake and alert
PSY3	I feel at choice in what thoughts I give attention to
PSY4	I can focus even in highly distracting situations
PSY5	I am able to maintain a positive outlook
	Factor 3: Emotional vitality
EMO1	I am aware of my emotional state
EMO2	I can influence my emotions when needed
EMO3	I have the energy I need to manage my stress
EMO4	I find time to relax and replenish my energy
EMO5	I have the emotional stamina to face problems

### Subjective Vitality Scale

Participants were asked to self-report their vitality through the six questions on the Subjective Vitality Scale (SVS). The SVS was first developed by Ryan and Frederick (1997) as a seven question scale and applied in 40 studies (Ryan and Frederick, 1997). The SVS was developed “to narrowly reflect a positive feeling of having personal energy” (Ryan and Frederick, 1997, p. 559) and the scale consists of a 7-point Likert scale from “not at all true” to “very true”. Bostic et al. (2000) re-evaluated the construct validity and utility of the SVS through structural equation modeling and found greater validity by removing the one negatively worded question from the scale (Bostic et al., 2000). The updated six-question SVS utilizes the inclusion of correlated error and thus has better goodness of fit indices than the longer version of SVS (GFI = 0.94 vs. 0.97) (Bostic et al., 2000).

### PsyCap PCQ-12

The Psychological Capital Questionnaire (12 items; PCQ-12) is the short version of the Psychological Capital Questionnaire (PCQ-24) (Luthans and Youssef-Morgan, 2017). The PCQ-12 consists of 12 items measuring hope, self-efficacy, resilience, and optimism. The scale uses a six-point Likert scale ranging from “I strongly disagree” to “I strongly agree”.

### Leader Vitality Using Cantril’s Ladder

Participants were directly asked to rate their physical vitality, psychological vitality, and emotional vitality using Cantril’s ladder (Gallop, 2021). Participants received the following questions and definitions for each and then were asked to rate each area on a scale of 0 (worst possible) to 10 (best possible).

• Where on the ladder do you stand now in terms of the amount of physical vitality you have (e.g., physical energy available to oneself, physical aliveness)?
• Where on the ladder do you stand now in terms of the amount of psychological or mental vitality you have (e.g., mental energy to think clearly, focus, be alert, have flexible thinking, and create a positive outlook)?
• Where on the ladder do you stand now in terms of the amount of emotional vitality you have (e.g., energy available to oneself to regulate emotions effectively)?

## Results

### Exploratory Factor Analysis

SPSS (version 26) was utilized in analyzing the collected data for exploratory factor analysis. The final dataset included 175 participants. All survey questions were required to be answered. There were a total of 24 surveys that were not completed and removed from the database. The data passed Bartlett’s test of sphericity (*p* < 0.001) and the Kaiser-Meyer-Olkin measure (KMO = 0.92), confirming that the items were sampled adequately to proceed with factor analysis (Hair et al., 1995; Tabachnick and Fidell, 2007). Factors were extracted using the principal axis factoring (PAF) method and rotated obliquely (Fabrigar et al., 1999). We conducted Horn’s (1965) parallel analysis to determine the number of factors that should be retained. No items had weak factor loadings (less than 0.50) and therefore we retained all 15 items. We then performed a series of EFA on the remaining items, to determine questions that had factor loadings less than 0.60. Based on this criterion we eliminated four questions—PHY5: I feel well rested when I wake up in the morning; PSY4: I can focus even in highly distracting situations; EMO1: I am aware of my emotional state; EMO4: I find time to relax and replenish my energy. The final set of 11 questions with factor loadings higher than 0.60 is displayed in Table 2. The initial EFA showed two factors, one for physical vitality and one for the combination of psychological and emotional vitality. Table 2 illustrates the items, item means, standard deviations, and factor loadings for the 11 items. The internal consistency of the 11 item LVS was high, with Cronbach’s alpha = 0.91.

**TABLE 2:** Exploratory factor analysis: items, means, standard deviations, and factor loadings of the LVS.

				Factor loadings	
	Factor Name and Items	M	SD	1	2
	**Factor 1: Physical vitality**				
PHY1	I drink water throughout the day	5.69	1.43		0.64
PHY2	I regularly eat healthy	5.32	1.52		0.65
PHY3	I incorporate movement into my day	5.67	1.41		0.87
PHY4	I have the physical stamina to do the things I want to do in my life	5.35	1.47		0.68
	**Factor 2: Psychological vitality**				
PSY1	I feel alive and vital	5.54	1.25	0.69	
PSY2	I nearly always feel awake and alert	4.95	1.38	0.73	
PSY3	I feel at choice in what thoughts I give attention to	5.41	1.33	0.73	
PSY5	I am able to maintain a positive outlook	5.85	1.02	0.82	
	**Factor 3: Emotional vitality**				
EMO2	I can influence my emotions when needed	5.46	1.11	0.76	
EMO3	I have the energy I need to manage my stress	5.29	1.31	0.74	
EMO5	I have the emotional stamina to face problems	5.75	1.10	0.76	

The factors demonstrated good internal consistency, with the physical vitality at a Cronbach’s alpha = 0.82 and the psychological and emotional vitality factor at a Cronbach’s alpha = 0.91. Factor correlations were 0.69 (see Table 3). The two factors explained 56% of the total variance observed in the LVS, indicating the retained items’ strength. Overall, these results provide initial evidence of construct validity.

**TABLE 3:** Exploratory factor analysis: matrix of factor correlations of the LVS.

	Factor	1	2
1	Physical Vitality	1.00	0.69
2	Psychological and Emotional Vitality	0.69	1.00

### Confirmatory Factor Analysis

**FIGURE 2:**
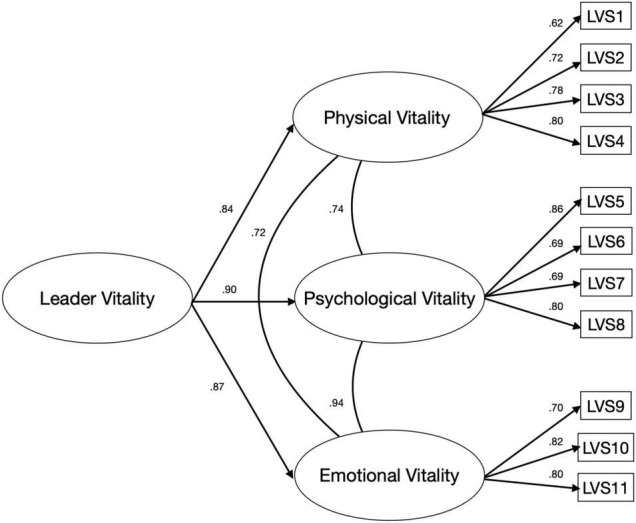
Graphical representation of a three-factor model with a higher-order factor.

R (version 1.3.1093) was then utilized in analyzing the collected data for confirmatory factor analysis. The data set showed adequate skewness and kurtosis, and histograms confirmed normal distributions. Scatterplots between the LVS and criterion variables were linear, with no specific patterns, ensuring homoscedasticity. CFA was performed using the maximum likelihood estimation procedure. First, we tested the two-factor model confirmed through exploratory factor analysis. Subsequently, we compared the two-factor model fit with a three-factor model with a higher-order factor as originally hypothesized. The two-factor model demonstrated good fit indices [*X*^2^ = 71.54, df = 44, CFI = 0.96, TLI = 0.95, SRMR = 0.06, RMSEA = 0.07; RMSEA 90% CI = (0.04, 0.10)]; CFI exceeded the 0.95 cutoff; SRMR was less than suggested 0.06 cutoff, RMSEA was less than 0.08 for adequate fit (Hu and Bentler, 1999; MacCallum and Austin, 2000; Kline, 2015). All items loaded significantly on the latent variables, with coefficients ranging from 0.67 to 0.85. The two subscales were highly intercorrelated at 0.75. We then assessed the hypothesized three-factor model with a higher-order factor, separating the psychological and emotional vitality factors. The subscales loaded with coefficients ranging from 0.62 to 0.82 (see Figure 2). The fit indices demonstrated a better model fit than the two-factor model [*X*^2^ = 65.85, df = 41, CFI = 0.96, TLI = 0.95, SRMR = 0.05, RMSEA = 0.07; RMSEA 90% CI = (0.04, 0.10)]. The subscales were highly intercorrelated, ranging from 0.72 to 0.94. Table 4 summarizes the model fit indices for both models. Because both the two-factor model and the three-factor model demonstrated good model fit, the two models were further examined. AIC score (5,510.20 vs. 5,509.36) and model comparison showed that data fit better on the three-factor model. However, the TLI difference was not statistically significant (< 0.01), indicating that the model difference is negligible (Gignac, 2007). In this case, we favored the three-factor model with a higher order factor because of the better fit with the theoretical perspective of vitality. Thus, we concluded that a three-factor model with a higher-order factor best supports the concept of vitality from both statistical and theoretical considerations.

**TABLE 4:** Confirmatory factor analysis of the LVS.

	*X* ^2^	*df*	CFI	SRMR	TLI	RMSEA	90%CI	AIC
Two-factor model	71.54	44	0.961	0.060	0.951	0.070	0.038, 0.099	5510.22
Three-factor model	65.85	41	0.964	0.046	0.952	0.069	0.036, 0.099	5509.36

*N = 175.*

*Rlative indexes: CFI = comparative fit index.*

*SRMR = standardized root mean square residual.*

*TLI = the Tucker–Lewis Index.*

*RMSEA = root-mean-square error of approximation.*

*AIC = the Akaike Information Criteria.*

### Convergent Validity Tests

We hypothesized that the LVS would be positively correlated with both the SVS and the SF-36 Health Survey vitality questions. We examined the Pearson correlation coefficient to test this hypothesis. Correlations among the LVS and the existing vitality scales were positive and significant (see Table 5).

**TABLE 5:** Convergent validity of the LVS.

	LVS	SVS	SF-36
LVS	1	0.77**	0.74**
SVS	0.77**	1	0.64**
SF-36	0.74**	0.64**	1

*N = 175.*

**Correlation is significant at the 0.01 level (1-tailed).

Another test of convergent validity was to evaluate the LVS factors against the three item Cantril’s Ladder scale for physical vitality, psychological vitality, and emotional vitality. The results show significant relationships across all factors with the strongest correlations between the LVS emotional factor and the ladder emotional vitality question (*r*^2^ = 0.78, *p* < 0.01), the LVS psychological factor and the ladder psychological vitality question (*r*^2^ = 0.70, *p* < 0.01), and the LVS physical factor and the ladder physical vitality question (*r*^2^ = 0.67, *p* < 0.01), (See Table 6).

**TABLE 6:** Convergent validity of the LVS factors and Cantril’s ladder vitality questions.

	LVS Physical	LVS Psychological	LVS Emotional	Ladder Physical	Ladder Psychological	Ladder Emotional
LVS Physical	1	0.61**	0.59**	0.67**	0.37**	0.45**
LVS Psychological	0.61**	1	0.78**	0.60**	0.70**	0.70**
LVS Emotional	0.59**	0.78**	1	0.54**	0.67**	0.78**
Ladder Physical	0.67**	0.60**	0.54**	1	0.53**	0.48**
Ladder Psychological	0.37**	0.70**	0.67**	0.53**	1	0.73**
Ladder Emotional	0.45**	0.70**	0.78**	0.48**	0.73**	1

*N = 175.*

**Correlation is significant at the 0.01 level (1-tailed).

A final evaluation was done to determine whether the LVS was unique from measures of Life Satisfaction and PsyCap. There is a significant correlation between the LVS and Life Satisfaction and PsyCap, but the LVS shows to be a unique construct (See Table 7).

**TABLE 7:** LVS correlations with life satisfaction and PsyCap.

	LVS	SWLS	PsyCap
LVS	1	0.56**	0.57**
LS	0.56**	1	0.46**
PsyCap	0.57**	0.46**	1

*N = 175.*

**Correlation is significant at the 0.01 level (1-tailed).

Analysis was done to compare female versus male participants, part-time versus full time employees, and managerial/leadership status differences for the LVS. There were no significant differences found between any of these comparisons.

## Discussion

The results of study 1 suggest that the Leader Vitality Scale (LVS) is a promising new way to measure leader vitality. We found in this study that the LVS is comprised of three unique factors, physical vitality, psychological vitality, and emotional vitality. The three factors appear to be distinct and to form a higher order construct of leader vitality. We also found that the LVS was related to the other more general vitality scales that we administered to provide some evidence for convergent validity.

## Study 2

Study 2 was conducted to further test the validity of the LVS with leaders in US based companies and the relationships between vitality, well-being, and psychological capital.

### Participants and Procedures

A total of 92 leaders ranging from manager to CEO were sampled from companies across the United States. The sample of leaders was collected through email recruitment and snowball sampling techniques. Of the sample, 48% were female (*n* = 44) and 50% were male (*n* = 46) and 2% preferred not to answer (*n* = 2). The participants’ ages ranged from 18 to 70 + years, with the highest number of respondents being between 41 and 55 years (*n* = 48). In terms of education, 80% of the participants possessed a bachelor’s degree or higher (*n* = 74). Regarding race/ethnicity, the majority self-identified as White/Caucasian (75%, *n* = 69), followed by 7% African American/Black (*n* = 6), Multiracial (5%, *n* = 5), Hispanic/Latino (5%, *n* = 5), Asian (4%, *n* = 4), preferred not to say (2%, *n* = 2) and Other/Unknown (*n* = 1). Eighty six percent of participants had been in their role for more than 1 year (*n* = 79). The participants’ roles are shown in Table 8.

**TABLE 8:** Leader roles.

	*n*	%
C-Suite	25	27
Senior Vice President/Vice President	23	25
Senior Director/Director	21	23
Manager	23	25

### Procedure

Participants completed a 75-question survey that included the 11 question Leader Vitality Scale (LVS), the six question Subjective Vitality Scale (SVS) (Ryan and Frederick, 1997), the Satisfaction with Life Satisfaction Scale (SWLS) (Diener et al., 1985), the Psychological Capital PCQ-12 (Luthans and Youssef-Morgan, 2017), the Positive Functioning at Work Scale (PF-W) created by Donaldson and Donaldson (2021b) and the Positive and Negative Affect Schedule (PANAS) scale (Watson et al., 1988). Demographic information was collected at the end of the survey, including age, gender, marital status, ethnicity, education level, and role tenure. All questions in the survey were required to be answered. Participants did not receive any compensation for completing the survey.

### Additional Measures

#### Building Blocks of Well-Being

Leaders were assessed through the Positive Functioning at Work Scale (PF-W) which measures the building blocks of work related well-being based on PERMA + 4 (Donaldson and Donaldson, 2021b; Donaldson et al., 2022). The PF-W integrates nine building blocks of well-being and positive functioning including positive emotions, engagement, relationships, meaning, accomplishment, physical health, mindset, environment, and economic security. The PF-W consists of 29 questions and is a validated scale with an overall reliability (α = 0.94). The reliabilities for each of the nine subcategories from acceptable (> 0.70) to excellent (> 0.90) (Howe, 1970): positive emotions (α = 0.93), engagement (α = 0.88), relationships (α = 0.90), meaning (α = 0.91), accomplishment (α = 0.81), physical health (α = 0.85), mindset (α = 0.86), environment (α = 0.76), and economic security (α = 0.84) (Donaldson and Donaldson, 2021b). Leaders were asked to report their nine building blocks of well-being and positive functioning of well-being based on a 7-point Likert scale (1 = *strongly disagree*, 2 = *disagree*, 3 = *somewhat disagree*, 4 = *neither agree or disagree*, 5 = *somewhat agree*, 6 = *agree*, 7 = *strongly agree)*.

#### Positive and Negative Affect Schedule

The PANAS was developed by Watson et al. (1988) and is two 10-item mood scale that comprises both positive and negative affect words. The scale has been shown to be highly internally consistent and stable with alpha reliabilities ranging from 0.86 to 0.90 for positive affect and from 0.84 to 0.87 for negative affect (Watson et al., 1988). The total score is calculated by finding the sum of the 10 positive items, and then the 10 negative items with scores ranging from 10 to 50 for both sets of items. For the total positive score and negative score, a higher score indicates more the positive or negative affect. PANAS has been widely and frequently used and has been validated in several languages, and it has shown excellent psychometric properties in the general population (Díaz-García et al., 2020).

## Results

SPSS (version 27) was utilized in analyzing the collected data for correlation analysis. The final dataset included 92 participants. All survey questions were required to be answered. There was a total of 57 surveys that were not completed and removed from the database. The data set showed adequate skewness and kurtosis, and histograms confirmed normal distributions. Analysis was done to compare gender and leadership level for LVS and there were no significant differences in gender or in leadership level. Scatterplots between the LVS and other scale variables were linear. Correlations among the LVS and the SVS were tested again for convergent validity and again showed a significant positive relationship (*r*^2^ = 0.86, *p* < 0.01). The internal consistency of the 11 item LVS was also high again, with Cronbach’s alpha = 0.89. There are also significant correlations between the LVS and Life Satisfaction, PF-W, PANAS and PsyCap (See Table 9).

**TABLE 9:** LVS correlations with Life Satisfaction, PF-W, PANAS, PsyCap.

	LVS	SWLS	PF-W	PANAS +	PANAS−	PsyCap
LVS	1	0.53**	0.76**	0.63**	−0.57**	0.47**
SWLS	0.53**	1	0.60**	0.41**	−0.48**	0.41**
PF-W	0.76**	0.60**	1	0.59**	−0.48**	0.58**
PANAS +	0.63**	0.41**	0.59**	1	−0.30**	0.54**
PANAS-	−0.57**	−0.48**	−0.48**	−0.30**	1	−0.39**
PsyCap	0.47**	0.41**	0.58**	0.54**	−0.39**	1

*N = 92.*

**Correlation is significant at the 0.01 level (1-tailed).

A further breakdown of the nine building blocks of PERMA + 4 and the correlations to the LVS are presented in Table 10 showing that the LVS is most highly correlated with physical health, then accomplishment, followed by relationships, environment, positive emotions, meaning, perception of financial security, and mindset with no significant correlation to engagement.

**TABLE 10:** LVS correlations PERMA + 4 pathways.

	Positive Emotions	Engagement	Relationships	Meaning	Accomplishment	Health	Mindset	Financial security	Environment
LVS	0.50**	−0.02	0.52**	0.26**	0.57**	0.73**	0.21*	0.30**	0.52**

*N = 92.*

**Correlation is significant at the 0.01 level (1-tailed).

*Correlation is significant at the 0.05 level (1-tailed).

A Bartlett’s test between Study 1 and Study 2 was run and results show there are no significant differences between Study 1 and Study 2 for the LVS or the factors of the LVS. A power test was also run and suggested these findings were not simply due to sample size.

## Discussion

Study 2 further confirmed that the LVS is a valid scale using data from a sample of top-level leaders within US based companies. The LVS was strongly related to PERMA + 4 (measured by the PF-W; Donaldson and Donaldson, 2021b) and positive emotions with an inverse relationship to negative emotions (measured by the PANAS; Watson et al., 1988). The LVS also showed to be highly related to 8 of the 9 building blocks of PERMA + 4. Furthermore the LVS is highly related to other measures used in positive psychology research including satisfaction with life (as measured by the SWLS; Diener et al., 1985) and psychological capital (as measured by PCQ-12; Luthans and Youssef-Morgan, 2017).

### General Discussion

We found in both study 1 and 2 that the LVS was highly related to leader well-being (as measured by the SWLS; Diener et al., 1985) and psychological capital (as measured by PCQ-12; Luthans and Youssef-Morgan, 2017) in the direction we would expect if the LVS was a valid measure of leader vitality. In study 2, we found the LVS was also significantly related to the nine PERMA + 4 building blocks of well-being and positive functioning. Our findings suggest that LVS has great promise for helping researchers develop a deeper understanding of the ways leader vitality can be defined and measured in future positive psychology research in the workplace. Previous measures of vitality have been not included all three important factors that the LVS encompasses. The LVS gives researchers and practitioners a more expanded way to research and evaluate the three factors of vitality including antecedents and outcomes of the individual factors. If our findings are replicated and extended in future research, it is possible that the LVS will provide more clarity to the opposite end of the burnout spectrum. Understanding how to measure the important resource of vitality can potentially help practitioners build it in a way that protects leaders from burnout, and enhances their leadership capacity, well-being, and positive functioning at work and beyond. The LVS can also be an important tool in leader development to help leaders think about their vitality and measure changes to this important resource over time.

The LVS could also be further validated and used in work to study the positive leadership framework recently proposed by Cameron (2021). This framework hypothesizes a leader’s ability to build positive relational energy with followers energizes them to create extraordinary results for their organization. That is, positive leaders encourage, empower, energize, and enhance the well-being and positive functioning of others. They are “positive energizers” in the workplace that create positive relational energy and heightened the level of psychological resourcefulness of their followers (Owens et al., 2016; Dutton et al., 2020; Cameron, 2021). The LVS could provide a new way to evaluate whether having physical, psychological, and emotional energy available to self can provide a foundation for more capacity for positive relational energy as well as positive leadership behaviors (e.g., actively listening, expressing gratitude, building trust, motivating others, creating meaning and purpose).

### Strengths and Limitations

The present study was able to provide empirical evidence that the LVS could be an effective way to measure leader vitality in future positive psychology research conducted in the workplace. The findings were based on a broad sample of leaders across companies and industries. The pattern of findings suggests the LVS could be an important measure for advancing our understanding of positive organizational psychology and specifically leader and follower well-being and optimal positive functioning (Donaldson and Chen, 2021; Donaldson et al., 2021b). We view these first empirical studies of the LVS as promising and encourage future researchers to overcome some of this study’s limitations and extend the empirical research in this important area of positive psychology.

First, it seems important to study leader vitality and well-being in larger and more diverse samples across multiple countries. Although we recognize the challenges of recruiting large numbers of diverse leaders for workplace well-being research, the time, effort, and resources needed to do this could have major long-term payoffs for future workplace well-being research and interventions. Second, the cross-sectional nature of the findings of this study are limited, and future longitudinal research could help us better understand the LVS and how leader vitality, well-being, and positive functioning interact over time. Finally, we recognized this first study of the LVS did not account for mono-method and self-report bias (Donaldson and Grant-Vallone, 2002; Donaldson and Donaldson, 2021a; Donaldson et al., 2021b). Ackerman et al. (2018) found that these limitations are somewhat common in related research on well-being. Nevertheless, we recommend future studies focus on ruling out these potential threats to validity by using more than self-report measures of leader vitality, well-being, and positive functioning. Future research is needed to see how self-reports of leader vitality converge or diverge from other measures like informant reports or physiological measures.

## Conclusion

Leaders face unique challenges and demands on their energy resources and need to maintain their own vitality and well-being in order to meet the requirements of their work and personal lives (Bruning et al., 2021). Vitality can provide a foundation of energy resources to a leader to serve at their full capacity and protect the leader’s resources from burnout. This study provides a new tool for measuring overall vitality and three sub-factors of physical vitality, psychological vitality, and emotional vitality. We are hopeful that over time the LVS will become a useful measure for assessing leader vitality, and for developing a better understanding of leader vitality, well-being, positive functioning at work, and positive organizational psychology 2.0 (see Donaldson et al., 2022).

## Data Availability Statement

The raw data supporting the conclusions of this article will be made available by the authors, without undue reservation.

## Ethics Statement

The studies involving human participants were reviewed and approved by Internal Review Board at Claremont Graduate University. The patients/participants provided their written informed consent to participate in this study.

## Author Contributions

JS and SD co-authored the manuscript together. Both authors equally contributed to the scale development and validation.

## Conflict of Interest

The authors declare that the research was conducted in the absence of any commercial or financial relationships that could be construed as a potential conflict of interest.

## Publisher’s Note

All claims expressed in this article are solely those of the authors and do not necessarily represent those of their affiliated organizations, or those of the publisher, the editors and the reviewers. Any product that may be evaluated in this article, or claim that may be made by its manufacturer, is not guaranteed or endorsed by the publisher.
